# The effect of previous acetabular fractures on total hip arthroplasty outcomes: A matched-controlled study protocol

**DOI:** 10.1097/MD.0000000000022210

**Published:** 2020-09-18

**Authors:** Lihong Wang, Pengfei Li, Jiangcui Kou, Changqing Hu

**Affiliations:** Department of surgical operating room, Harrison International Peace Hospital, Hebei Province, China.

**Keywords:** acetabulum fracture, complication, Harris Hip Score, protocol, total hip arthroplasty

## Abstract

**Background:**

Total hip arthroplasty (THA) is a safe and mature surgical method for the treatment of post-traumatic arthritis and end-stage degenerative osteoarthritis. The cohort study regarding the outcomes of THA following acetabulum fracture is sparse. Therefore, we carried out this present retrospective paired cohort study to study the long-term outcomes of patients receiving THA after the acetabular fracture versus patients receiving THA for the primary osteoarthritis.

**Methods:**

Patients with posttraumatic arthritis who received the initial THA after open reduction and internal fixation of acetabular fractures or patients with end-stage degenerative osteoarthritis were included in our study. A retrospective review of patients who receiving the primary total hip arthroplasty in the same institution from 2008 to 2015 was conducted. This present retrospective cohort research was authorized via our hospital institutional review committee. The patients in cohort group were matched 2:1 with the patients in study group according to following criteria: body mass index (±3 points), and age at THA time (±3 years), sex, as well as the score of American Society of Anesthesiologists (±1 point). The measure of primary outcome was the improved Harris Hip Score. Secondary outcomes included surgery time, hip range of motion, revision, complications (infection, loosening, polyethylene wear, dislocation, wound complications, deep vein thrombosis, or pulmonary embolism).

**Results:**

It was assumed that there is a remarkable difference in postoperative outcomes between the 2 groups.

**Trial registration:**

This study protocol was registered in Research Registry (researchregistry5921).

## 1 Introduction

With an aging population, the prevalence and burden of all osteopenic fractures, especially hip fractures, continue to be a concern.^[1–3]^ Specifically, in patients over the age of 60, in the past 25 years, the incidence rate of acetabular fractures has increased,^[4]^ and the incidence of acetabular fractures in the elderly patients is the fastest growing subgroup.^[5,6]^ The complex anatomic area provides familiar concomitant injuries, management difficulties and long-term complications. Although the modern techniques of fracture management allow these fractures to approach anatomic reduction, there is still a risk of post-traumatic arthritis, which thus inevitably requires further total hip arthroplasty (THA).

THA is a safe and mature surgical method for the treatment of post-traumatic arthritis and end-stage degenerative osteoarthritis. It has been found that THA can effectively relieve pain, increase the motion range and the self-reported mobility and function of patients who are ineffective in the non-surgical treatments.^[7–10]^ When arthritis and necrosis of femoral head occur after trauma, THA is a reasonable rescue method. The residual acetabular defects, scar tissue, and the retention of internal fixation implants lead to the subsequent THAs, which is more complex than conventional THA. In addition, because of abnormal anatomical structure and the degree of bone stock loss after trauma, the results are not as good as THA conducted after the primary osteoarthritis.^[11–16]^

Although some former researches have assessed patients receiving THA after the acetabular fractures, however, most of them did not set up a control group, and the follow-up time was limited.^[17–23]^ The cohort study regarding the outcomes of THA following acetabulum fracture is sparse. Therefore, we carried out this present retrospective paired cohort study to study the long-term outcomes of patients receiving THA after the acetabular fracture versus patients receiving THA for the primary osteoarthritis. It was assumed that there is a remarkable difference in postoperative outcomes between the 2 groups.

## 2 Materials and methods

### 2.1 Patients

The inclusion criteria were patients with posttraumatic arthritis who received the initial THA after open reduction and internal fixation of acetabular fractures or patients with end-stage degenerative osteoarthritis; patients over 18 years old who can cooperate with us in treatment and the observation after operation; complete demographic data and follow-up data were available. Exclusion criteria contained the patients were attempted or already pregnant, with a alcoholism history, cognitive impairment, or whether the patient received bilateral THA at the same time.

### 2.2 Study design

A retrospective review of patients who receiving the primary total hip arthroplasty in the same institution from 2008 to 2015 was conducted. This present retrospective cohort research was approved by the Institutional Review Board in Harrison International Peace Hospital (2020090) and has been registered in research registry (researchregistry5921). The patients in cohort group were matched 2:1 with the patients in study group according to following criteria: body mass index (BMI) (±3 points), and age at THA time (±3 years), sex, as well as the score of American Society of Anesthesiologists (ASA) (±1 point).

### 2.3 Surgery technique

All the surgeries in the 2 cohorts were conducted via an experienced author under vertical laminar airflow through utilizing the modified Hardinge lateral approach. All the operations were carried out under the condition of general anesthesia in laminar flow operating room. In all the cases, the tourniquet was utilized, the operative hip was prepared and covered with a routine sterile manner. The 2 femoral components were the cementless titanium-alloy stems with porous coating of proximal titanium and were conventionally conducted. The 2 acetabular components were cement-free modular hemispherical cups with holes for the auxiliary screw fixation and a porous titanium coating.

### 2.4 Postoperative management

The postoperative drainage continued for 1 to 2 days until the flow was less than 30 ml. All the patients was given the same standardized postoperative multimodal pain m. All patients underwent the same protocol of postoperative rehabilitation program. The complete weight bearing assisted by crutches was allowed for 6 weeks after the surgery. Each patient was assessed radiologically and clinically at 6 weeks, 3 months, 6 months, and 12 months after operation, followed by an annual evaluation.

### 2.5 Outcomes

The BMI, ASA classification, and demographics of patients were retrospectively recorded from their electronic patient notes. The measure of primary outcome was the improved Harris Hip Score. The Harris Hip Score was initially utilized to assess the post-traumatic arthritis treatment, but it is now extensively utilized in any osteoarthritis of hip, at the same time, it has been found to respond to these patients. The highest score was 100 (the optimal function), covering pain (1 item, from 0–44 points), activities and function (7 items, from 0–47 points), and the motion range and no deformity (3 items, from 0–9 points). Secondary outcomes included surgery time, hip range of motion, revision, complications (infection, loosening, polyethylene wear, dislocation, wound complications, deep vein thrombosis, or pulmonary embolism). All the related complications, operation time, and the demand for the revision surgery were acquired from the EMR. All the data were verified independently via detailed hospital surgical reports, the clinical records, and anesthesia records. The Harris Hip Score and motion range were acquired via physiotherapist specialist before and after the arthroplasty (Tables 1 and 2).

**Table 1:**
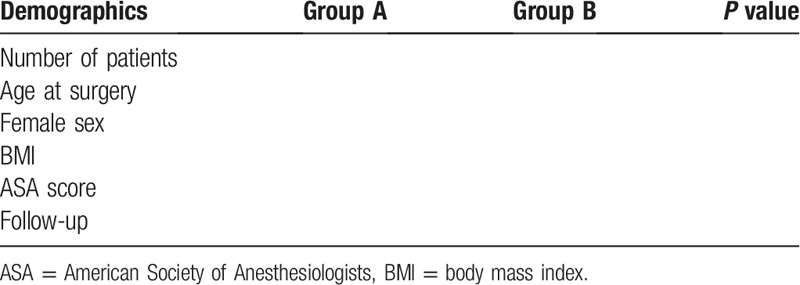
Patient baseline demographics.

**Table 2:**
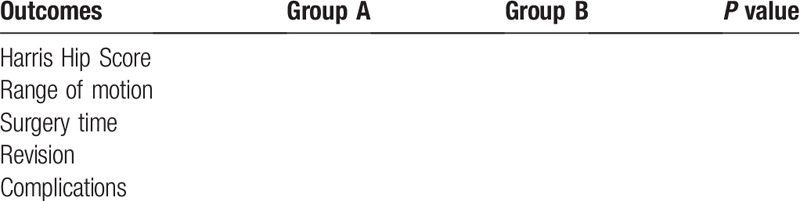
Clinical outcomes.

### 2.6 Statistical analysis

The Chi-Squared test and Students unpaired *t* test were respectively utilized for the comparisons of the qualitative variables and qualitative outcomes between the 2 groups. The program of SPSS software for Windows was applied to conduct all the statistical analyses (version 22; IBM Corp., Armonk, NY, USA). The *P* value <0.05 was regarded as statistically significant.

## 3 Discussion

Acetabular fractures are the complex hip joint injuries, and its morbidity is high. The recovery of joint consistency has a significant impact on the treatment effect. Nevertheless, the development of post-traumatic osteoarthritis can even appear after the anatomical reconstruction. The development of post-traumatic arthritis causes the functional impairment and pain stimulation. In this case, the in-depth surgery in the form of THA is recommended. Several authors have suggested that the initial hip arthroplasty may be regarded as an alternative to THA in cases where long-term hip joint longevity after the hip reconstruction is not guaranteed, allowing patients to move at an early stage rather than having to undergo a secondary THA.^[24–26]^

Nevertheless, the understanding of the outcome of THA treatment for previous acetabular fractures is presently limited. The literature regarding the outcome of THA following acetabulum fracture is sparse. Therefore, we carried out this present retrospective paired cohort study to study the long-term outcomes of patients receiving THA after the acetabular fracture versus patients receiving THA for the primary osteoarthritis. It was assumed that there is a remarkable differences in postoperative outcomes between the 2 groups. The limitations of our present research include the inherent limitations in any retrospective cohort research, including the observation bias and possibility of selection. In addition, although we conducted matching research according to gender, age, BMI and ASA score, there may be other preoperative characteristics that we can control for that may cause alternative outcomes.

## Author contributions

Lihong Wang and Pengfei Li conceived, designed, and planed the study. Lihong Wang, Pengfei Li, and Jiangcui Kou are recruiting the study participants and performing the interventions. Changqing Hu supervised the study. Lihong Wang, Pengfei Li, and Jiangcui Kou will interpret and analyze the data. Lihong Wang drafted the manuscript. Pengfei Li critically revised the manuscript for important intellectual content. All authors have full access to the manuscript and take responsibility for the study design. All authors have approved the manuscript and agree with submission.

**Conceptualization:** Pengfei Li.

**Data curation:** Lihong Wang, Pengfei Li.

**Formal analysis:** Lihong Wang, Pengfei Li.

**Funding acquisition:** Changqing Hu.

**Investigation:** Jiangcui Kou.

**Methodology:** Lihong Wang, Jiangcui Kou.

**Project administration:** Changqing Hu.

**Resources:** Changqing Hu.

**Software:** Jiangcui Kou.

**Supervision:** Changqing Hu.

**Validation:** Jiangcui Kou.

**Visualization:** Jiangcui Kou.

**Writing – original draft:** Lihong Wang.

**Writing – review & editing:** Pengfei Li.

## Abbreviations

Abbreviations: ASA = American Society of Anesthesiologists, BMI = body mass index, THA = total hip arthroplasty.
